# Dietary iron does not impact the quality of life of patients with quiescent ulcerative colitis: an observational study

**DOI:** 10.1186/1475-2891-12-152

**Published:** 2013-11-23

**Authors:** Zoe Tolkien, Dora IA Pereira, Laura Prassmayer, Emily Fitt, Gerda Pot, Simon M Greenfield, Jonathan J Powell

**Affiliations:** 1 MRC Human Nutrition Research, Elsie Widdowson Laboratory, Cambridge, UK; 2 Diabetes & Nutritional Sciences Division, School of Medicine, King’s College London, London SE1 9NH, UK; 3 Gastroenterology Department, QE2 Hospital, East and North Hertfordshire NHS Trust, Welwyn Garden, UK

**Keywords:** Dietary iron, Quality of life, Ulcerative colitis, Patients

## Abstract

**Background:**

In animal models, excess luminal iron exacerbates colonic inflammation and cancer development. Moreover, in inflammatory bowel disease (IBD) patients with mild to moderate disease activity dietary fortificant iron intake is inversely related to quality of life. Here we sought to determine whether dietary iron intakes were also related to quality of life in IBD patients in remission.

**Methods:**

Forty eight patients with ulcerative colitis (UC), 42 of which had quiescent disease during this observational study, and 53 healthy control subjects completed quality of life questionnaires and 7-day food diaries. For comparative analysis, 34/group were matched and the linear relationship between dietary iron intakes (total, haem, non-haem or fortificant) and EuroQol quality of life measures was investigated. For UC patients the linear relationship between dietary iron intakes and the scores from the disease specific inflammatory bowel disease questionnaire (IBDQ) was also considered.

**Results:**

The intake of dietary iron, and its various sub-fractions, were not associated with quality of life (EuroQol) in patients with quiescent disease or in healthy control subjects. The picture was similar for the 42 quiescent UC patients when disease-specific IBDQ was used. However, the 6 patients who relapsed during the study again showed an inverse association between IBDQ and dietary iron intake (*p* = 0.03).

**Conclusions:**

Our data suggest that dietary iron does not impact on quality of life in quiescent UC patients but support that, once the disease is triggered, luminal iron may be a permissive factor for exacerbation of disease activity resulting in lower quality of life.

## Background

Anaemia due to iron deficiency is prevalent amongst patients with inflammatory bowel disease (IBD) [1-3]. Although prescription of oral iron therapy is common practice it may, on occasions, undermine the intended benefits of therapy by simultaneously aggravating symptoms and thereby possibly reducing quality of life in these patients [4]. For patients who are not in stable remission, aggravation of gastrointestinal symptoms could occur through the overproduction of harmful reactive oxygen species in the colon, catalysed by a surplus of unabsorbed supplemental iron [5]. Indeed, in murine models of IBD, high levels of dietary iron increase inflammation and colonic lipid peroxidation, and induce mucosal damage [6,7]. Furthermore, IBD patients have an increased risk of developing colorectal cancer [8] and, worryingly, recent murine studies have suggested strong promotion of intestinal tumorigenesis with an increase in luminal iron concentration [9,10].

Reactive (or ‘free’) iron in the intestinal lumen has not been characterised. However, even for supplementation with soluble ferrous (Fe(II)) iron salts most of the iron will not remain in the lumen in a readily reactive form. Oxidation, and then oxo-hydroxide precipitation, in the gut lumen will greatly limit the ‘free’ iron. Thus free reactive iron in the gut lumen, that can exacerbate inflammation, may occur at only a low concentration. In light of this, the potential adverse effects of iron at normal (or near normal) fortified dietary iron intake levels have been investigated albeit data are still scarce. Nonetheless, all suggest a potential detrimental effect of iron. An intervention study in healthy, non-anaemic middle-age men using flour fortified with 30 mg Fe/kg as ferrous sulphate for more than 1 year has shown a significant increase in systemic biomarkers of oxidative stress such as superoxide dismutase and glutathione peroxidase [11]. Zimmermann *et al.* have reported significant changes towards a more detrimental gut microflora profile in children supplemented with electrolytic iron-fortified biscuits for 6 months [12]. To our knowledge, the only human study in IBD is our own recently published data indicating a detrimental effect of dietary iron, in particular from Fe-fortified products, on the quality of life of IBD patients with mild-moderately active disease [13]. The aim of the observational study presented herein was to determine the effect of dietary iron intakes on the quality of life of ulcerative colitis (UC) patients with inactive (i.e. quiescent) disease for at least 1 month. In particular, and unlike in our previous work, we used a single, defined and larger (n = 50) IBD population (quiescent UC) and used the most robust, albeit resource-consuming, dietary assessment technique, namely 7-day food diaries. As a reference group we also studied healthy controls and they, for analysis, were matched to subjects with UC.

## Methods

### Study design

This observational study was conducted according to the guidelines laid down in the Declaration of Helsinki and all procedures involving human subjects were approved by the Cambridge Central Research Ethics Committee (11/EE/0312). Written informed consent was obtained from all subjects.

Fifty ulcerative colitis (UC) patients were recruited from gastrointestinal outpatient clinics at Lister Hospital (Stevenage), Addenbrooke’s Hospital (Cambridge) and Queen Elizabeth II (QE2) Hospital (Welwyn Garden City), all in the United Kingdom. Fifty healthy control subjects were recruited from the general population in the Cambridgeshire area. Controls were matched with cases for gender and age (within 5 years).

All participants completed a 7-day, estimated food diary and a EuroQol EQ-5D-5 L health-related quality of life questionnaire. In addition, patients with ulcerative colitis completed a disease specific quality of life questionnaire, namely the Inflammatory Bowel Disease Questionnaire (IBDQ), as well as a disease activity form, i.e. the Simple Clinical Colitis Activity Index (SCCAI).

### Patients

Eligible case patients included those aged 18 – 80 with a confirmed diagnosis of ulcerative colitis by histological and/or radiological techniques and with disease in remission at the time of enrolment in the trial (minimum of 3 months). Clinical remission was defined as SCCAI < 5 [14]. Exclusion criteria were active UC (i.e. SCCAI ≥ 5), anaemia of any kind (haemoglobin < 12 g/dL), other chronic disease, any form of iron therapy, proton-pump inhibitor therapy, eating disorders, pregnancy or breast-feeding. Patients who had received over-the-counter iron supplementation, erythropoiesis stimulating agents (EPO) or blood transfusions in the previous 28 days were also excluded.

### Controls

The control group was recruited from the general population and consisted of healthy subjects aged 18–80 years old with no history of chronic disease, gastrointestinal disease or anaemia. Further exclusion criteria were as above.

### Quality of life questionnaires

The EuroQol EQ-5D-5 L is self-administered and is comprised of five dimensions of health: mobility, self-care, usual activities, pain/discomfort, and anxiety/depression [15,16]. Each dimension has five levels: (1) no problems, (2) slight problems, (3) moderate problems, (4) severe problems, (5) inability/extreme problems. An ‘index value’ from 0–1 is generated using the Crosswalk Index Value Calculator [17,18]. In addition, the questionnaire captures a self-rating of health status by a visual analogue scale (VAS) which is limited at 100 (best imaginable health) and 0 (worst imaginable health).

The McMaster IBDQ is specific to inflammatory bowel disease and has been extensively validated [19-21]. This questionnaire is more sensitive to changes in quality of life for patients with inflammatory bowel disease compared to general health questionnaires, and is able to display changes in disease activity for patients with ulcerative colitis or Crohn’s disease [22]. The McMaster IBDQ consists of 32 questions designed to assess four different domains: emotional, bowel, social and systemic. Each question is scored on a seven point Likert scale (1, worst function; 7, best function) and the final score ranges from 32 to 224. Higher scores indicate a better quality of life. The questionnaire was self-administered.

### Disease activity

The Simple Clinical Colitis Activity Index (SCCAI) was used to determine whether patients remained in disease remission following referral by their gastrointestinal consultant and to provide a formal IBD disease activity score upon enrolment in the study [23]. The SCCAI consists of 4 questions regarding bowel movements, one question regarding general wellbeing and an area to report any extra-colonic features (one point per manifestation). A score of 5 or more indicates disease relapse [14].

### Dietary assessment

All subjects completed a 7-day estimated diet diary which has been validated with data from the European Prospective Investigation into Cancer (EPIC) [24]. Subjects were asked to record everything they had to eat or drink and to use household measurements to describe portion size. Subjects also collected all food packaging to increase the accuracy of the dietary data entry. Data from the diet diaries were recorded using in-house programme DINO (Diet In Nutrients Out) which is an all-in-one dietary recording and analysis system written in Microsoft Access [25]. DINO was developed at MRC Human Nutrition Research and incorporates the Department of Health’s Nutrient Databank [26] and McCance and Widdowson’s Composition of Foods series [27]. Quality was assured by a random 10% check of all entered diaries. After data entry, each participant’s mean energy and nutrient intake over the seven diary days were calculated.

Additionally, as before [13] fortificant iron intakes were calculated separately as detailed below and comprised foods that are fortified with iron on a voluntary basis by manufacturers (e.g. breakfast cereals), as well as foods containing white flour (e.g. bread), which is restored with iron by law. Fortificant iron was calculated as follows: (i) for food products which are fortified with iron to varying levels at the discretion of the manufacturer, e.g. breakfast cereals, the level of ‘natural’ (i.e. not added) non-haem iron was estimated based on the ingredient information using the McCance and Widdowson food composition tables [27], and this value was then subtracted from the total iron content declared by the manufacturer to estimate fortificant iron content; and (ii) for other foods containing white wheat flour, which is fortified with iron as a requirement under the UK Bread and Flour Regulation 1995, fortificant iron was estimated by considering the content of white flour per food portion and using the standard ‘restoration’ formula of 1.65 mg Fe/100 g white flour to derive the content of fortificant iron per food portion. All values for the different categories of dietary iron were calculated as total daily intakes for each subject.

### Statistical analysis

A sample size of 50 subjects per group was calculated to give 88% power to detect a 2 point decrease in the disease specific quality of life score for each 1 mg increase in iron intake. This sample calculation was based on our previous data [13] demonstrating a similar effect size in a linear regression analysis with residual standard error of 26 for the quality of life score and standard deviation for iron intake of 5.25.

Unless otherwise stated, all statistical analysis was performed using GraphPad Prism 6 for Windows (GraphPad Software, San Diego, California, USA). Normality of the data was tested with the D’Agostino and Pearson omnibus normality test. Comparisons between case patients and controls in terms of diet and quality of life were made using the Mann–Whitney nonparametric test. Results are presented as means with standard deviations (SD). Significance of associations between the intakes of the different fractions of dietary iron and quality of life were assessed using simple linear regression analysis (Gaussian distribution of X-data not assumed) and multiple linear regression (with SPSS Statistics 21). Comparison of the slopes for the respective associations of quality of life and dietary iron intakes, between UC patients with disease in remission and those with disease in relapse, were performed by analysis of covariance (ANCOVA). Statistical significance was assumed at *p* < 0.05.

## Results

**Figure 1:**
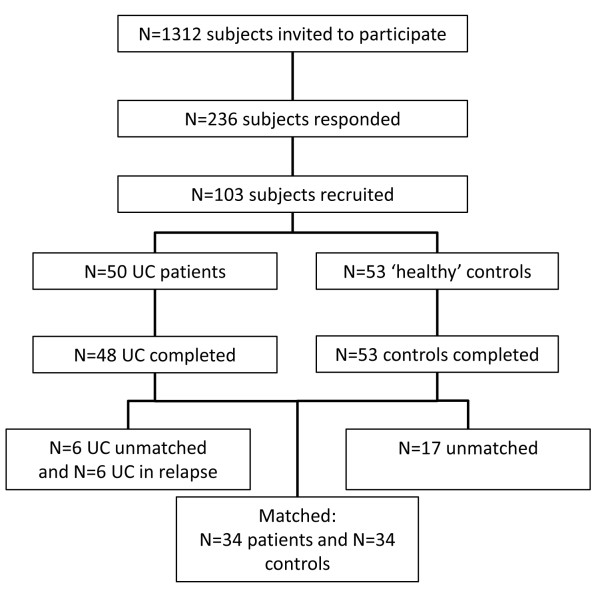
**Study participants flow chart.** From the 236 initially responding subjects 133 decided not to take part when they received more detail of study. The 2 patients that did not complete the study did so for reasons unrelated to the study.

A total of 103 subjects were enrolled in the study (Figure 1). Of the UC cases that completed the study 26 were male (54%) and 22 were female (46%) with a mean age (range) of 50 (23–78) years. Of the healthy controls 26 were male (49%) and 27 were female (51%) with a mean age (range) of 41 (20–76) years. The differences between groups were not statistically significant. Six UC patients relapsed during the study and were, therefore, excluded from the main analysis. Of the remaining UC patients 34 were matched for age (within 5 years) and gender with controls.

**Figure 2:**
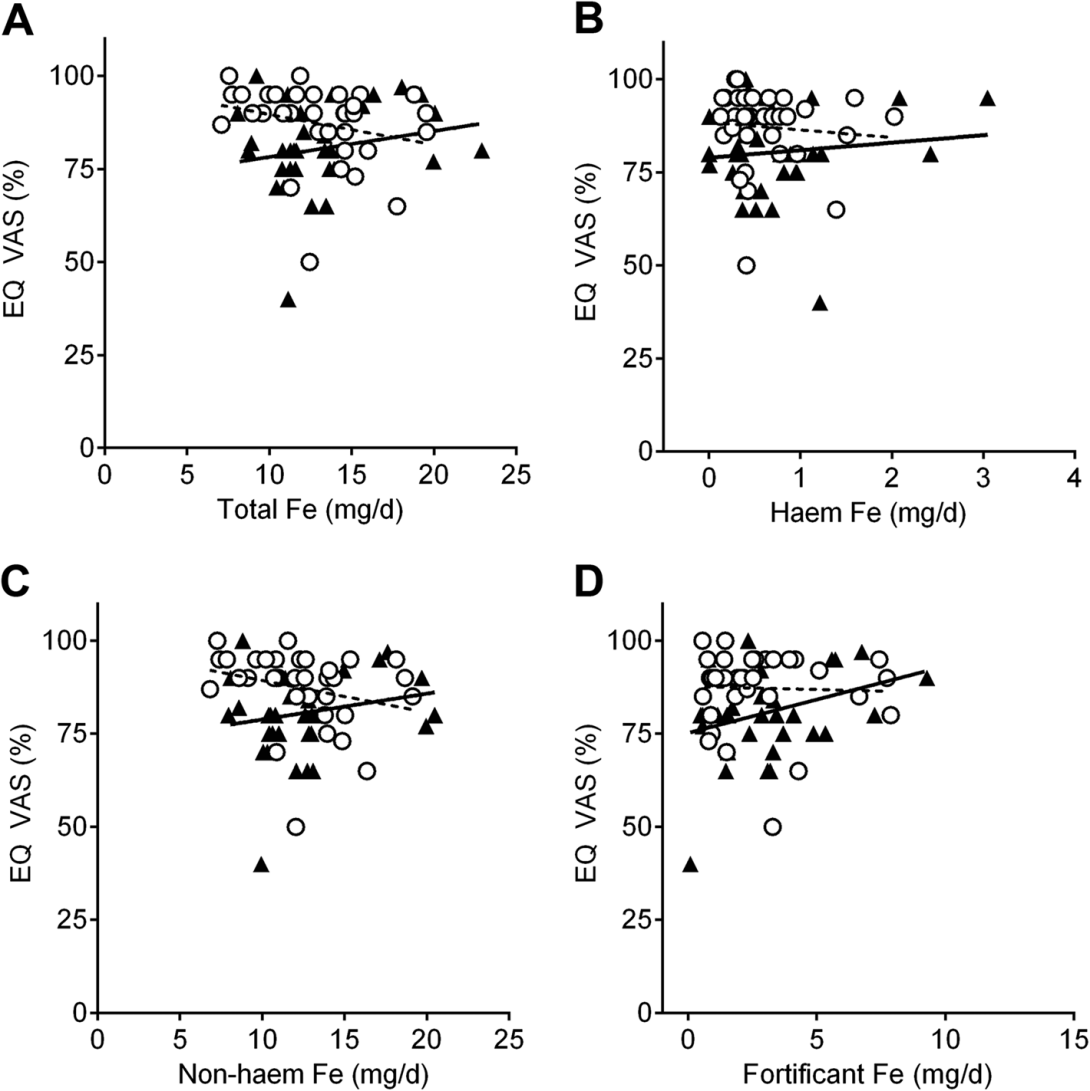
**Association between dietary iron intakes and quality of life measured with EuroQol VAS (EQ VAS%) in UC patients with quiescent disease (closed triangles and full black line) and matched ‘healthy’ controls (open circles and dashed black line). A**, total dietary iron; **B**, haem iron; **C**, non-haem iron; **D**, fortificant iron. There were no significant slope deviations from zero (linear regression) for both UC patients and matched controls (*p* > 0.05, n = 34 per group).

**Figure 3:**
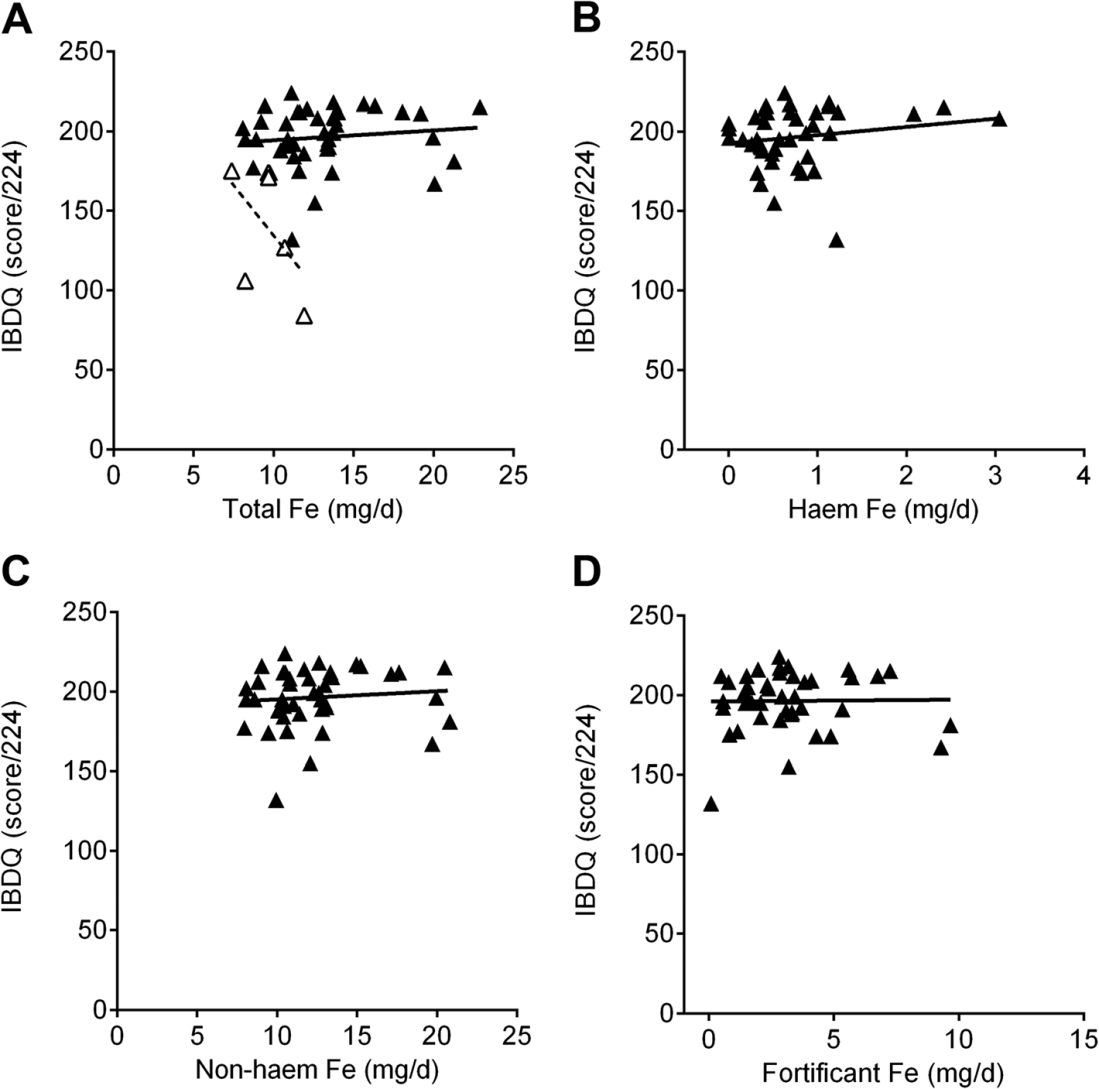
**Association between dietary iron intakes and quality of life measured with McMaster IBDQ for UC patients with quiescent (closed triangles and full black line, n = 42) or relapsed (open triangles and dashed black line, n = 6) disease activity. A**, total dietary iron; **B**, haem iron; **C**, non-haem iron and **D**, fortificant iron. *p* = 0.029 (for total dietary iron, panel A) for the comparison of the slopes for the respective linear associations using analysis of covariance (ANCOVA). For relapsing patients, further analysis was not considered appropriate for the sub-fractions of dietary iron given the very small study numbers.

The mean EuroQol VAS score for the UC patients with quiescent disease (i.e. in remission) was 81% (95% CI = 76–85), which was significantly lower than that of matched controls (87%: 95% CI = 84–91) (*p* = 0.007). Dietary intake of all ‘forms’ of iron (i.e. total, haem, non-haem and fortificant) had no statistical significant linear association with the EuroQol VAS score in the matched cases and controls (Figure 2). Similarly when the unmatched datasets were compared in their entirety (i.e. 42 UC patients in remission and 53 controls) there was again no difference between the two groups and no association with dietary iron intakes. The same was true when using the EuroQol index value (Additional file 1: Figure S1) or the IBDQ score (patients only) as quality of life measures (Figure 3). Multiple linear regression, with fortificant iron, haem iron and ‘natural’ (i.e. non-fortificant, dietary-derived) non-haem iron as independent variables (predictors) and the quality of life measure (i.e. EQ VAS, EQ index or IBDQ score) as the dependent variable, also showed no association. Furthermore, there were no statistically significant differences for the intake of all ‘forms’ of dietary iron between cases and controls in the matched study group. A summary of the dietary intake of the main nutrients in the matched cases and controls is presented in Additional file 2: Table S1.

**Figure 4:**
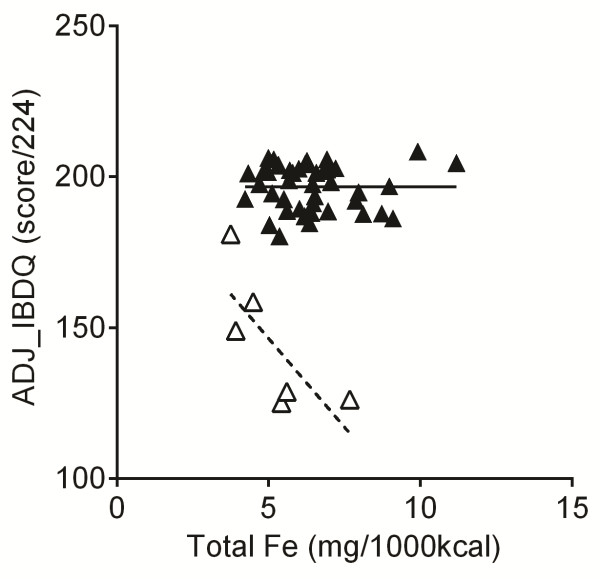
**Association between dietary iron intakes and adjusted IBDQ scores for UC patients with quiescent (closed triangles and full black line, n = 42) and relapsed (open triangles and dashed black line, n = 6) disease activity.** IBDQ scores are predicted values adjusted for age and gender (entered as covariates in the linear regression model). Dietary iron is expressed as iron density values. *p* = 0.0001 for the comparison of the slopes for the respective linear associations using analysis of covariance (ANCOVA).

Although not part of the above main analysis we briefly considered the six patients who relapsed during the study period. Notably, these patients had significantly lower dietary intakes of total and non-haem iron compared to patients in remission (i.e. the cases in the matched study group), even though total daily energy intakes and general diet composition were comparable (Table 1). Unsurprisingly, the relapsing patients also had lower quality of life scores (Table 1) and, splitting the analysis into the four IBDQ domains (i.e. bowel, systemic, emotional and social) showed that, while all domains were associated with relapse of disease, bowel and emotional function scores dropped the most (Table 2). As observed previously [13] for the 6 patients who relapsed there was an inverse trend towards lower IBDQ scores (i.e. quality of life) with higher total dietary iron intakes (*p* = 0.029 for the comparison of the slopes of the linear associations for active and inactive disease, Figure 3). The significance of this difference was further strengthened when the IBDQ scores were adjusted for disease activity and total dietary iron was expressed corrected for daily energy intakes (*p* = 0.0001, Figure 4). Numbers were too small to consider subdividing the analysis for the different forms of dietary iron.

**Table 1:** Simple Colitis Clinical Activity Index score, quality of life scores and dietary intakes of main nutrients in UC patients in remission or relapse

	***Patients in remission (n = 42)***	***Patients in relapse (n = 6)***	
	**Mean (95% CI)**	**Mean (95% CI)**	*p*-value*
**Age**	51 (46–55)	44 (35–52)	ns
**SCCAI (score/19)**	2.3 (1.9-2.7)	7.3 (5.3-9.4)	<0.0001
**EQ VAS (%)**	82 (79–86)	58 (36–79)	0.002
**EQ index value**	0.90 (0.86-0.93)	0.76 (0.63-0.89)	0.005
**IBDQ (score 32–224)**	196 (190–202)	140 (98–181)	<0.0001
**Energy intake (MJ/d)**	8.71 (8.11-9.31)	8.13 (6.60-9.67)	ns
**Carbohydrates (g/d)**	246 (226–265)	247 (198–297)	ns
**Protein (g/d)**	82.0 (75.5-88.4)	67.0 (47.9-86.0)	ns
**Fat (g/d)**	77.7 (71.4-83.9)	80.9 (61.9-99.9)	ns
**Fibre (g/d)**	15.6 (14.0-17.2)	12.9 (10.8-15.1)	ns
**Zing (mg/d)**	11.4 (10.2-12.6)	11.1 (5.0-17.2)	ns
**Calcium (mg/d)**	1,059 (949–1,168)	1,003 (738–1,269)	ns
**Total Fe (mg/d)**	13.1 (12.0-14.2)	9.6 (7.9-11.3)	0.006
**Haem Fe (mg/d)**	0.73 (0.54-0.92)	0.41 (0.18-0.64)	ns
**Non-haem Fe (mg/d)**	8.5 (7.7-9.4)	5.9 (4.7-7.2)	0.007
**Fortificant Fe (mg/d)**	3.5 (2.7-4.3)	3.2 (1.8-4.7)	ns

*Abbreviations*: *SCCAI* simple colitis clinical activity index, *EQ* EuroQol EQ-5D-5 L, *EQ VAS* EuroQuol Visual Analogue Scale score, *IBDQ* McMaster inflammatory bowel disease questionnaire, *MJ* megajoules (equals 1000 kilojoules), *Fe* iron, *CI* confidence interval. *Mann–Whitney nonparametric test. Statistical significance was considered at *p* < 0.05. ns, not significant.

**Table 2:** Individual scores of the IBDQ domains

	***Patients in remission (n = 42)***	***Patients in relapse (n = 6)***	
	**Mean (95% CI)**	**Mean (95% CI)**	*p*-value*
**IBDQ Bowel (10 questions)**	62 (60–64)	41 (29–54)	<0.0001
**IBDQ Systemic (5 questions)**	28 (26–30)	23 (15–30)	ns
**IBDQ Emotional (12 questions)**	72 (70–75)	49 (35–64)	<0.0001
**IBDQ Social (5 questions)**	34 (33–35)	27 (18–35)	0.0004

*Abbreviations*: *IBDQ* McMaster inflammatory bowel disease questionnaire, *CI* confidence interval. *Mann–Whitney nonparametric test. Statistical significance was considered at *p* < 0.05. ns, not significant.

## Discussion

Our previous data have shown that elevated dietary iron intakes, especially fortificant iron intakes, are associated with a reduced quality of life in IBD patients with mild-moderate disease activity [13]. This implies that during relapse, luminal iron levels become an attributable factor in the severity of inflammation. This is supported by supplementation data in rodent models over the past 10 years as well as more recent work showing that depletion of luminal iron alleviates gut inflammation in IBD models [6,7,28].

The aim of the present study was to investigate if, in the *absence* of inflammation (i.e. in inactive (quiescent) disease), this might still be the case. Our results show that, in the absence of clinical disease activity (i.e. SCCAI < 5), there was no measurable linear association between dietary iron intakes and quality of life which is consistent with earlier preliminary findings [13].

However, even though the numbers are very small (n = 6), our data in UC patients that relapsed after enrolling in the study, and developed mild-moderate active disease, again showed a negative association between dietary iron intake and the disease-specific quality of life score (IBDQ). This very small number of patients would not justify further sub-analysis but we do note that, for all UC patients studied herein, the IBDQ scores gave a good correlation with EuroQol scores, especially EQ VAS(%) (Additional file 3: Figure S2). The small dataset also showed that mean dietary iron intakes were lower in the UC patients with mild-moderate active disease compared with their counterparts in remission and this is in agreement with previous studies in IBD [29]. It suggests that the disease state, itself, modifies patient food choice.

The use of 7-day food diet diaries and carefully matched control data means that we can suggest confidently, from this work that dietary iron does not have any clinically meaningful effect on quality of life in UC patients with inactive disease. However, the picture appears different in the presence of existing mucosal inflammation: some degree of disease activity, even if mild (SCCAI ≥ 5), may be sufficient to allow a detectable association between quality of life and dietary iron intake (Figure 3A and [13]). Moreover, this appears to be driven by fortificant iron [13]. Taken together, there may also be important resonances between our studies (here and [13]) and the recent work of Radulescu *et al.*[9] where dietary iron was shown to be a permissive factor for colon cancer development in murine models.

## Conclusions

We propose that luminal free iron may be a contributing factor in the severity of colonic inflammation, once triggered through another means, in patients with UC. We do acknowledge that data from the robust observational study presented here only provide suggestion of associations and a more conclusive intervention study in patients with active disease is now required. If the association shown here and in our prior preliminary work [13] is confirmed, then, whether it operates through the well described reactive oxygen species-inducing nature of reactive iron in the colon, or through iron’s modulation of the microbiota [7], or indeed some other mechanisms(s) would warrant careful investigation, as would the possibility of intervention through sequestration of free luminal iron in the distal bowel.

## Competing interests

This work was supported by the Medical Research Council. ZT was in receipt of an MRC studentship. LP was in receipt of an ERASMUS studentship. The authors declare no conflict of interests.

## Authors’ contributions

The authors’ responsibilities were as follows: JJP, ZT and DIAP designed the study in consultation with SG. ZT carried out the study. ZT, LP, EF and GP carried out diet data entry and dietary assessment. ZT and DIAP carried out data analysis in consultation with JJP. All authors contributed to the preparation of the manuscript and have approved the manuscript. JJP, ZT and DIAP had primary responsibility for the final content of the manuscript.

## Supplementary Material

Additional file 1: Figure S1 Association between dietary iron intakes and quality of life measured with EuroQol index in UC patients with quiescent disease (closed triangles and full black line) and matched ‘healthy’ controls (open circles and dashed black line). A, total dietary iron; B, haem iron; C, non-haem iron; D, fortificant iron. There were no significant slope deviations from zero (linear regression) for both UC patients and matched controls (*p*>0.05, n=34 per group). Click here for file

Additional file 2: Table S1 Age, quality of life and dietary intakes of main nutrients in matched cases and controls. Click here for file

Additional file 3: Figure S2 Linear association between quality of life measured with the McMaster IBDQ and the generic EuroQol EQ-5D-5L. A, EQ VAS, EuroQuol Visual Analogue Scale score; B, EQ index value. The best-fit line is shown with the 95% confidence band and the *p*-value for the linear associations: n=48. Click here for file
