# Supplementary material for: Dietary iron does not impact the quality of life of patients with quiescent ulcerative colitis: an observational study
Source: Nutr J. 2013 Nov 23;12:152. doi: 10.1186/1475-2891-12-152 (PMC4222872; doi:10.1186/1475-2891-12-152)
Supplement: Additional file 2: Table S1 — Age, quality of life and dietary intakes of main nutrients in matched cases and controls. [file 1475-2891-12-152-S2.docx]

**Dietary iron does not impact the quality of life of patients with quiescent ulcerative colitis: an observational study.**

*Zoe Tolkien, Dora IA Pereira, Laura Prassmayer, Emily Fitt, Gerda Pot, Simon M Greenfield, Jonathan J Powell*

**Supplementary Information**

**Supplementary Table S1. Age, quality of life and dietary intakes of main nutrients in matched cases and controls.**

|  | ***Cases (n=34)*** | ***Controls (n=34)*** |  |
| --- | --- | --- | --- |
|  | **mean (95% CI)** | **mean (95% CI)** | ***p*-value^*^** |
| **Age** | 50 (45-55) | 45 (39-50) | ns |
| **Euroquol index value** | 0.90 (0.86-0.94) | 0.91 (0.87-0.96) | ns |
| **Energy intake (MJ/d)** | 8.7 (8.0-9.4) | 8.6 (8.0-9.3) | ns |
| **Carbohydrates (g/d)** | 240 (217-262) | 253 (228-279) | ns |
| **Protein (g/d)** | 83.1 (75.6-90.6) | 82.3 (74.0-90.6) | ns |
| **Fat (g/d)** | 78.4 (71.6-85.2) | 77.8 (70.2-85.4) | ns |
| **Fibre (g/d)** | 15.8 (14.0-17.5) | 17.4 (15.6-19.1) | ns |
| **Zinc (g/d)** | 11.2 (10.0-12.4) | 12.6 (9.4-15.9) | ns |
| **Calcium (g/d)** | 1,028 (908-1,147) | 1,116 (1,012-1,220) | ns |
| **Vitamin A (retinol equivalents) (µg/d)** | 1,081 (828-1,333) | 1,189 (924-1,455) | ns |
| **Vitamin E (α-tocopherol equivalent) (mg/d)** | 14.1 (8.0-20.2) | 10.7 (9.6-11.8) | ns |
| **Vitamin C (mg/d)** | 123.5 (58.4-188.7) | 134.2 (73.5-194.8) | ns |
| **Total Fe (mg/d)** | 13.2 (12.0-14.4) | 13.0 (11.8-14.1) | ns |

**Abbreviations: EQ**, EuroQol EQ-5D-5L; **CI**, confidence interval; **MJ**, megajoules (equals 1000 kilojoules); **Fe**, iron. *Mann-Whitney nonparametric test. Statistical significance was considered at *p*<0.05. ns, not significant.

**Supplementary Figure S1. Association between dietary iron intakes and quality of life measured with EuroQol index in UC patients with quiescent disease (closed triangles and full black line) and matched ‘healthy’ controls (open circles and dashed black line). A,** total dietary iron; **B,** haem iron; **C,** non-haem iron; **D,** fortificant iron. There were no significant slope deviations from zero (linear regression) for both UC patients and matched controls (*p*>0.05, n=34 per group).

**Supplementary Figure S2. Linear association between quality of life measured with the McMaster IBDQ and the generic EuroQol EQ-5D-5L. A**, EQ VAS, EuroQuol Visual Analogue Scale score; **B**, EQ index value. The best-fit line is shown with the 95% confidence band and the *p*-value for the linear associations: n=48.
